# *Bacillus amyloliquefaciens* SC06 in the diet improves egg quality of hens by altering intestinal microbiota and the effect is diminished by antimicrobial peptide

**DOI:** 10.3389/fnut.2022.999998

**Published:** 2022-11-01

**Authors:** Shujie Xu, Fei Wang, Peng Zou, Xiang Li, Qian Jin, Qi Wang, Baikui Wang, Yuanhao Zhou, Li Tang, Dongyou Yu, Weifen Li

**Affiliations:** ^1^Hainan Institute, Zhejiang University, Sanya, China; ^2^Key Laboratory of Molecular Animal Nutrition of the Ministry of Education, Key Laboratory of Animal Nutrition and Feed Science (Eastern of China) of the Ministry of Agriculture, Key Laboratory of Animal Feed and Nutrition of Zhejiang Province, College of Animal Sciences, Institute of Animal Nutrition and Feed Sciences, Zhejiang University, Hangzhou, China

**Keywords:** laying hen, *B. amyloliquefaciens*, antimicrobial peptide, cecum microbial abundance, short-chain fatty acids

## Abstract

This experiment investigated the effects of *Bacillus amyloliquefaciens* SC06 (BaSC06) and its combination with antimicrobial peptide (AMP) on the laying performance, egg quality, intestinal physical barrier, antioxidative status and immunity of commercial Jingbai strain laying hens. The results showed that BaSC06 significantly improved laying performance and egg quality of laying hens. However, there was a tendency to increase laying performance and decrease egg quality for the addition of AMP compared to the BaSC06 group. Also, both BaSC06 and its combination with AMP treatment increased length of microvilli and the content of tight junction protein in jejunum, and BaSC06 combination with AMP treatment is better than BaSC06 treatment alone. Compared to control, most of the serum antioxidant enzyme activities were significantly increased in the BaSC06+AMP group, the BaSC06 group only increased the activity of GSH-Px. Short-chain fatty acid analysis showed that BSC06 significantly increased the content of butyric, isobutyric and isovaleric acid in the cecum. However, the content of most of the short-chain fatty acids was even lower than that of the control group after the addition of AMP. Microbiota analysis showed that BaSC06 increased the absolute abundance of the butyrate-producing gut bacteria *Ruminococaaoeae UCG-005*, while the addition of AMP reduced the number of microorganisms detected and weakened the effect of BaSC06. BaSC06 acts as an anti-inflammatory agent by regulating the gut microbiota, and AMP further attenuates the immune response by reducing the number of gut microbes based on improved intestinal microbiota composition.

## Introduction

Eggs have an enormous economic value, are an excellent source of animal protein and have become an important consumer product worldwide due to their low cost (1). Egg production in quantity and quality to satisfy public demand is the main object of the commercial laying hen industry. In recent years, the use of humates, enzymes, probiotics, antimicrobial peptide and their combinations as feed additives to improve health status, performance traits and feed conversion ratio, has become common in animal nutrition (2, 3). These growth stimulations are superior to antibiotics because they have no negative effects on the consumer (4). Various research has shown that a combination of active ingredients can provide better effects than a single ingredient (5–7). Finding the right combination can improve the quality and quantity of eggs, which is essential for the laying hen industry.

*Bacillus amyloliquefaciens* SC06 is a probiotic isolated from soil, and our previous studies found that it can be used as an alternative to antibiotics to affect the intestinal epithelial barrier and immune function in piglets and broilers by modulating the intestinal flora (8, 9). According to reports, supplementing a basal chicken diet with *B. amyloliquefaciens* BLCC1-0238 can improve laying performance and egg quality by reducing stress responses, up-regulation of growth hormones, and supporting immunity in laying hens (10). *Bacillus amyloliquefaciens* B-1895 can improve egg production, quality of sperm production, quality/hatchery of eggs, and slow down the reproductive aging of hens (11). However, the effects of *B. amyloliquefaciens* SC06 on laying hen's performance and healthy status remain unclear.

Antimicrobial peptides (AMPs) are low molecular weight proteins with broad-spectrum antimicrobial and immunomodulatory activity against infectious bacteria, viruses and fungi (12). Among the most widespread AMPs in nature, cationic α-helical AMPs are capable of disrupting the cytoplasmic membrane of bacteria, leading to cell death through osmotic shock (13). The most important group of AMPs are cecropin, magainin, the human cathelicidin LL-37, their derivatives and proline rich antimicrobial peptides (14). In particular, Cecropins are used as pharmaceutical and feed additives (15). It is a natural cationic AMP produced by silkworms that has been shown to have high levels of antimicrobial activity and is considered as a worthwhile peptide antibiotic (16).

As mentioned above, the function of AMP is mainly to kill microorganisms in the gut, while the role of BaSC06 is to regulate the gut microorganisms through which they perform their functions. Therefore, we conjecture that the addition of AMP may enhance the effect of probiotics. Therefore, the objective of this study was to evaluate the effects of *B. amyloliquefaciens* SC06 and the combination of BaSC06 with AMP on laying hens performance, egg quality, intestinal health and gut microbiota.

## Materials and methods

### Bacterial strains and culture conditions

*Bacillus amyloliquefaciens* SC06, previously isolated from the soil, Hangzhou, China, was cultured in Luria-Bertani (LB) medium and incubated at 37°C for 18 h. The incubated bacterial liquid was mixed with 40% glycerol at 1:1 and stored at −80°C. Before use, the stored bacterial liquid was inoculated into fresh LB medium and incubated at 37°C for 18 h.

### Diet preparation and feeding trial

The experiment was carried out in accordance with the Chinese guidelines for animal welfare and approved by the Animal Welfare Committee of Animal Science College, Zhejiang University. In this experiment, a total of 270 commercial laying hens of Jingbai strain at the age of 202 days with the similar performance were randomly allotted to three dietary treatment groups. Each group had six replicates with 15 laying hens. The control group was fed a basal diet. The BaSC06 group was fed a basal diet supplemented with *B. amyloliquefaciens* SC06 (5 × 10^8^ CFU/kg). The AMP+BaSC06 group was fed a basal diet supplemented with 100 mg/kg AMP and *B. amyloliquefaciens* SC06 (5 × 10^8^ CFU/kg). The antimicrobial peptide used was laboratory-prepared cecropins AD and was administered according to the recommended dose. The sequence encoding cecropin AD is KWKLFKKIEKVGQRVRDAVISAGPAVATVAQATALAK and is secreted by the *Bacillus subtilis* expression system. The basic corn-soybean meal diets were formulated to meet or exceed the nutritional requirements for laying hens calculated according to The National Research Council recommended (Table 1). Use staggered three-layer cages, each independent cage puts 5 laying hens, 1 week before entering the chickens, the cage is disinfected according to the usual procedures. Sixteen hours of light a day, natural light supplemented by artificial light. The feeding experiment lasts for 6 weeks. The first week is a preliminary experiment, and the next 5 weeks are a formal experiment. During the preliminary experiment, we observed the laying rate of laying hens and adjusted each group so that there is no statistical difference in laying rate between groups. During the entire experiment period, they were free to eat and drink, and were fed twice a day at 7:30 in the morning and 15:00 in the afternoon. Immunization was carried out according to the routine immunization program.

**Table 1:** Ingredients and nutrient contents of the basal diet (As-fed basis).

**Items**	**Value**
**Ingredients (%)**	
Corn	57.00
Soybean meal, 46% CP	24.00
Wheat middling	5.50
Microalgal DHA powder	1.00
Limestone	9.00
Dicalcium phosphate	1.00
Salt	0.30
DL-methionine	0.12
Lysine-HCl	0.08
Premix^a^	2.00
Total	100.00
**Nutrient level** ^**b**^	
Metabolizable energy, Mcal/kg	2.65
Crude protein, %	16.43
Lysine, %	0.89
Methionine, %	0.40
Cysteine + methionine, %	0.75
Calcium, %	3.62
Total phosphorus, %	0.56
Available phosphorus, %	0.35
Selenium, mg/kg	0.02

a The premix provided the following per kg of the diet: iron, 60 mg; copper, 10 mg; manganese, 80 mg; zinc 80 mg; iodine 0.3 mg; vitamin A, 12,500 IU; vitamin D3, 4,000 IU; vitamin K3, 2 mg; thiamine, 1 mg; riboflavin, 8.5 mg; calcium pantothenate, 50 mg; niacin acid, 32.5 mg; pyridoxine, 8 mg; folic acid, 5 mg; B12, 5 mg; choline chloride, 500 mg; phytase, 1,000 IU.

b The nutrient levels were calculated values.

### Sample preparation

At the end of the experiment (42 d), all laying hens were deprived of feed for 12 h. Six laying hens (one hen per replicate) were selected and marked from each treatment group randomly, weighed and blood samples were collected before slaughter from the wing vein using 5-mL vacuum blood tubes. Blood samples were and placed at room temperature and centrifuged at 3,000× g for 30 min. The serum samples were collected and stored at −20°C until analysis. In addition, laying hens were euthanized to enable the collection of tissues. The jejunum was ligated and separated, the middle part of the intestine segment was taken and fixed in 2.5% buffered glutaraldehyde for transmission electron microscopy (TEM). The rest of the jejunum segment and the whole caecum as well as the intact ovary of laying hens was sampled, snapped frozen in liquid nitrogen and then stored at −80°C for further analysis.

### Production performance assay

Egg production and mass were recorded daily (at 8:00), and feed consumption was recorded weekly on a replicate basis (6 replicates per dietary treatment) to calculate the laying rate, average daily egg mass, average daily feed intake, and feed conversion ratio (feed/egg: g/g) as follows: laying rate (LR) (%) = Total number of eggs/laying hens number/days (d) × 100; feed conversion ratio (FCR) = Total feed consumption (g)/total egg weight (g); average daily egg mass (ADEM) (g/hen/day) = Total egg mass (g)/laying hens number/ days (d); average daily feed intake (ADFI) (g/hen/day) = [total final feed intake (g) – total initial feed intake (g)]/days (d)/laying hens number.

### Egg quality determination

Eight eggs from each group were randomly collected at the end of the experiment to determine the egg quality. The egg shape index is calculated by measuring the transverse and longitudinal diameters of eggs with vernier calipers. Eggshell thickness was measured (without shell membrane) with a caliper at 3 different points (air cell, sharp end, and any side of the equator) and estimated by the average of the 3 different thickness measurements from each egg. Egg weight, eggshell strength, albumen height, yolk color, and haugh unit (HU) were measured by using a digital egg tester (DET-6000, Nabel Co., Ltd., Kyoto, Japan). Then weigh the yolk to calculate the yolk ratio. Egg shape index = Longitudinal diameter of the egg (cm)/Transverse diameter of the egg (cm); yolk ratio (%) = Egg yolk mass (g)/Egg mass (g) × 100.

### Serum biochemical parameters

The activities of lactic dehydrogenase (LDH) (No. A020-1-2), myeloperoxidase (MPO) (No. A044-1-1), glutamic pyruvic transaminase (ALT) (No. C009-2-1), glutamic oxalacetic transaminase (AST) (No. C010-1-1), total antioxidant capacity (T-AOC) (No. A015-1-2), superoxide dismutase (SOD) (No. A001-1-1), catalase (CAT) (No.A007-1-1) and glutathione peroxidase (GSH-Px) (No. A005-1-2) as well as the concentration of total cholesterol (TC) (No. A111-1-1), total protein (TP) (No. A045-2-2), albumin (ALB) (No. A028-1-1), blood urea nitrogen (BUN) (No. C013-1-1), glucose, triglyceride, uric acid (UA) (No. C012-1-1) and malondialdehyde (MDA) (No. A003-1-2) in the serum were determined by using commercial kits based on manufacturer's guidelines (Jiancheng Bioengineering Institute, Nanjing, Jiangsu, China).

### ELISA assay

The concentrations of progesterone (Prog) (No. H089), follicle-stimulating hormone (FSH) (No. H101-1-2) and luteinizing hormone (LH) (No. H206-1-2) were determined by using an enzyme-linked immunosorbent (ELISA) kits (Jiancheng Bioengineering Institute, Nanjing, Jiangsu, China) according to the manufacturer's instruction.

### Transmission electron microscopy

After fixation in 2.5% glutaraldehyde buffer, jejunum tissue was washed 3 times every 15 min in 0.1 M cold phosphate buffer. The tissue was fixed in 0.1% osmium tetroxide (OsO_4_) cold buffer for 2 h, and then washed with phosphate buffer. After rapid dehydration in successively increasing ethanol solutions (30, 50, 70, 95, and 100%), the tissues were transferred to a 1:1 mixture of epoxy propane and epoxy aldehyde resin. After embedding, ultrathin sections (60–100 nm) were cut with an LKB Nova ultra-slicer (Leica Microsystems, Buffalo Grove, IL) and stained with uranyl acetate. Electron microscopic images of intestinal mucosal cells and microvilli were taken by transmission electron microscope (JEOL, Tokyo, Japan) at 80 kV. The length of the microvilli was determined using the length measurement tool of ImageJ. The images were invert colors to remove background interference before quantitative tight junctions were made.

### RNA extraction and RT-qPCR

Ileal and hepatic total RNA was isolated using Trizol reagent (Takara, Dalian, China), according to the manufacturer's protocol. The ratio of absorbance at 260 nm to that at 280 nm was calculated for each sample. The complementary DNA was synthesized using the HiScript^®^IIReverse Transcriptase kit (Vazyme), and real-time PCR was performed using the SYBR Premix Ex Tap (Vazyme). The PCRs were run on the StepOnePlus™ Real-Time PCR System. Amplification cycles involved initial heating at 95°C for 30 s, followed by 40 cycles of 95°C for 10 s, 60°C for 30 s. Each mRNA level was expressed as its ratio to β-actin mRNA, and gene expression was calculated using the 2-ΔΔCt method. All primer sequences for target genes are listed in Table 2.

**Table 2:** Sequences of oligonucleotide primers used for RT-qPCR.

**Gene name**	**Primer's sequence**	**Accession number**
P53-F	CGCCGTGGCCGTCTATAAG	NM_205264.1
P53-R	GTACAGTCAGAGCCCACCTCG	
Caspase-3-F	ACTCTGGAAATTCTGCCTGATGACA	NM_204725.2
Caspase-3-R	CATCTGCATCCGTGCCTGA	
IL-1β-F	CGACATCAACCAGAAGTGCTT	NM_204524.2
IL-1β-R	GTCCAGGCGGTAGAAGATGA	
IL-10-F	TGCTGGATGAGTTTAAGGGGAC	NM_001004414.2
IL-10-R	CCCATGCTCTGCTGATGACT	
IFN-γ -F	CAACGACACCATCCTGGACA	NM_205427.1
IFN-γ -R	ATCCGGTTGAGGAGGCTTTG	
MCT1-F	AGCAGCATCCTGGTGAACAAG	Zhange et al. (17)
MCT1-R	AGGCACCCACCCACGAT	
IL-8-F	AGCACTCATTCTAAGTTCATCC	NM_205498
IL-8-R	CCAAGCACACCTCTCTTCCATC	
IL-13-F	GGTCCCTTGCAATGACACCA	NM_001007085
IL-13-R	GTGATGAGGGGCTCGTAGTC	
NF-κB-F	CGAACTCCGTGACGATTCCC	NM_204413
NF-κB-R	ATACCGAAATCGGAAGCCCC	
NLRP3-F	CTACGGCCGTCTACGTCTTC	Wang et al. (18)
NLRP3-R	GGCCAAAGAGGAATCGGACA	
Caspase-1-F	GTGACCATTCTTTCGCACGG	Wang et al. (18)
Caspase-1-R	TTGGCAGTTAGCTCAGCACA	
ASC-F	CTGGAGATGTGGTTTGGCCT	Wang et al. (18)
ASC-R	TTGGTTCTTGACCATCCGCA	
β-actin F	TATGTGCAAGGCCGGTTTC	NM_205518.2
β-actin R	TGTCTTTCTGGCCCATACCAA	

### Microbial analysis

Microbial genomic DNA was extracted under sterile conditions from the cecal content of laying hens using the TIANamp Stool DNA Kit (Tiangen, Beijing, China) according to the manufacturer's instructions. The quality of extracted DNA was checked by agarose gel electrophoresis and spectrophotometric analysis. The V3–V4 region of the 16S rRNA gene was amplified using the primer pair 341F/805R, and sequencing was performed on MiSeq platform (Illumina Inc., San Diego, CA, USA). Sequences were filtered and clustered into operational taxonomic unit (OTU) with 97% similarity by QIIME software (version 1.9.1).

Alpha diversity (Goods, Ace, Chao1, Shannon, and Simpson) was calculated to reflect bacterial diversity and richness. Principal coordinate analysis (PCoA) which is based on binary euclidean and binary jaccard was performed to get principal coordinates and visualized from complex data. The relative abundance of microbiota was examined at different taxonomic levels. The histogram of linear discriminant analysis (LDA) distribution was implemented using LDA effect size analysis (LEfSe) software. The relative abundance of significant differences in phylum, class, order, and OTU levels was calculated by the one-way analysis of variance (ANOVA). The 16S rRNA gene sequencing information was analyzed by PICRUSt2 to predict biological functions and metabolic pathways (KEGG database) of the bacterial community of intestinal contents samples of laying hens.

### Analysis of short-chain fatty acids

Take 0.1 g cecal contents were vortex-mixed vigorously with 10 mL deionized water. After the mixture were centrifuged (12,000 rpm for 10 min), 500 μL aliquots of the supernatant were added to 100 μL of 25% (w/v) metaphosphoric acid and crotonic acid (internal standard). The mixed solution was filtered with a 0.22 μm mesh and was then employed to measure the concentrations of SCFAs by GC (GC-2010 plus, Shimadzu, Kyoto, Japan).

### Statistical analysis

All data was analyzed by one-way analysis of variance (ANOVA) and the contrast of means was performed using Tukey's multiple range test by SPSS software (SPSS Inc., Chicago, IL, USA). Results were expressed as means ± standard error of mean (SEM), and the values of *P* ≤ 0.05 was considered significant. Graphs were drawn using the GraphPad Prism 8.4.2 software.

## Results

### Laying performance

The effects of BaSC06 and BaSC06+ AMP on the laying performance of laying hens are shown Table 3. Compared with the control group, the laying rate of the two treatment groups have tendency to improve (*P* > 0.05). Additionally, the average egg weight in all two treatment groups increased significantly (*P* < 0.05), with group BaSC06+AMP, showing the greatest increase.

**Table 3:** Laying performance of laying hens fed different biological feed additives^1^.

**Item**	**Control**	**BaSC06**	**BaSC06+AMP**	**SEM**	***P*-value**
LR, %	87.43	88.76	89.90	0.75	0.167
Average egg weight (g)	57.50^b^	58.39^a^	58.60^a^	0.14	0.002
ADEM, g/hen/day	52.64	52.69	53.03	0.21	0.713
ADFI, g/hen/day	102.31	103.75	102.35		
FCR, g ^feed^/g ^egg^	2.07	2.02	1.95	0.026	0.079

1 Results are the mean of 6 replicates of 15 laying hens each.

LR, laying rate; ADEM, average daily egg mass; ADFI, average daily feed intake; FCR, feed conversion ratio.

a,b Value differences in the same row differ significantly (P < 0.05).

### Egg quality parameters

At the end of the feeding cycle, 1–2 fresh eggs were randomly selected from each replicate for egg quality testing. As shown in Table 4, BaSC06 significantly improved yolk color and yolk percentage of egg compared with control group (*P* < 0.05). However, these improvements became insignificant with the addition of AMP, this situation leads us to speculate that AMP may weaken the effect of BaSC06.

**Table 4:** Egg quality parameters of laying hens^1^.

**Item**	**Control**	**BaSC06**	**BaSC06+AMP**	**SEM**	***P*-value**
Egg-shaped index	1.31^a^	1.31^ab^	1.28^b^	0.06	0.027
Eggshell strength, kg/cm^2^	3.78	3.92	3.78	0.11	0.748
Albumen height, mm	6.96	7.49	8.08	0.24	0.187
Haugh unit	82.2	85.71	89.16	1.86	0.137
Yolk color	5.70^b^	6.50^a^	6.30^ab^	0.13	0.023
Eggshell thickness, mm	0.39	0.40	0.39	0.004	0.392
Yolk percentage, %	56.00^b^	58.82^a^	57.96^ab^	0.005	0.047

1 Results are the means of each group of 8 eggs.

a,b Value differences in the same row differ significantly (P < 0.05).

### Intestinal physical barrier function and biochemical parameters

**Figure 1:**
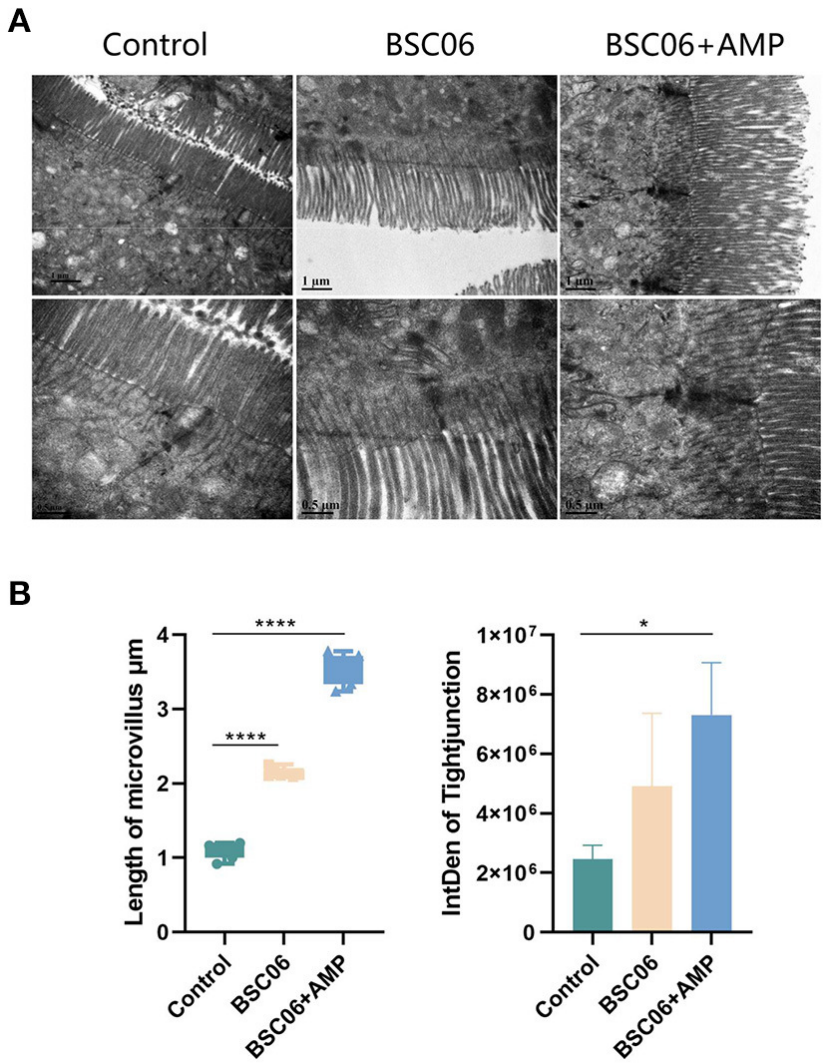
**(A)** Transmission electron micrographs of the jejunum microvilli in laying hens. **(B)** Quantitative analysis of microvilli length (*n* = 6) and tight junction (*n* = 3) based on TEM (BSC06: *Bacillus. amyloliquefaciens SC06*; BSC06+AMP: *Bacillus. amyloliquefaciens SC06* combined with antimicrobial peptide). Data are presented as the means ± SD for *n* = 12; *P < 0.05, ****P < 0.0001.

All of BaSC06 and AMP have protective effects on intestinal tract and the strong correlation is established between intestinal health and host health, we used TEM to observe the microstructure of the colon of laying hens (Figure 1A). Interestingly, BaSC06+AMP can significantly increase the length of microvilli (*P* < 0.0001) and the gray-scale value of tight junction protein and the effect is stronger than using BaSC06 alone (*P* < 0.05; Figure 1B). This result suggests that AMP still exerts its own protective effect, and the weakened effect on BaSC06 may be due to the conflict between their functions.

**Figure 2:**
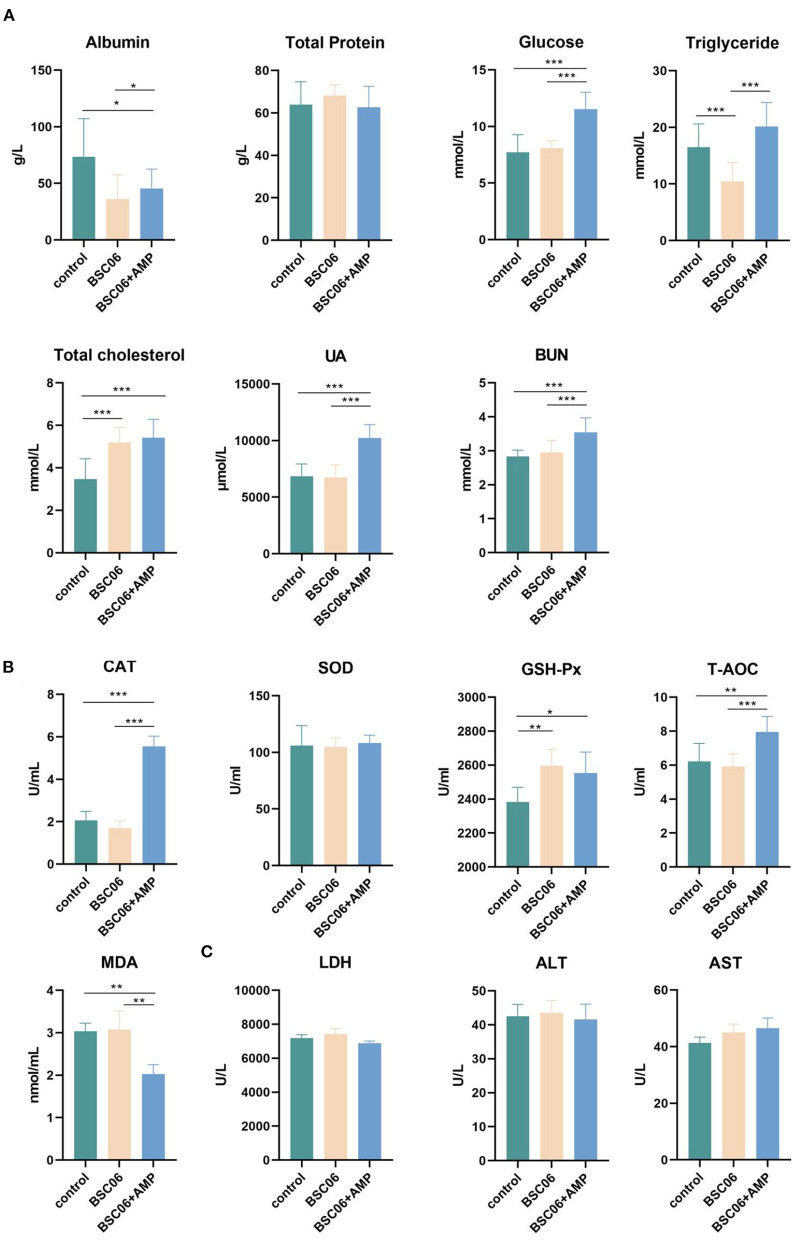
Blood biochemical parameters of laying hens. **(A)** Nutrition-related biochemical parameters. **(B)** Measurement of related indexes of antioxidant levels in serum. **(C)** Content of LDH, ALT and AST level in serum (*n* = 6). Data are presented as the means ± SD for *n* = 12; *P < 0.05, **P < 0.01, ***P < 0.001.

After drawing the conclusion of BaSC06 and AMP can play their respective roles in the intestinal tract of laying hens, we want to investigate the effect of both additives on the overall health of the host by measuring the regular biochemical parameters in serum. As shown in Figure 2A, BaSC06 and BaSC06+AMP significantly decreased serum albumin and increased content of total cholesterol (*P* < 0.05). Critically, the addition of AMP promoted the levels of glucose, UA, and BUN in the serum (*P* < 0.001), whereas, feeding BaSC06 alone had no such effect. Similarly, the addition of AMP can significantly improve CAT (*P* < 0.001) and total antioxidant capacity (T-AOC) (*P* < 0.01), and reduce MDA content (*P* < 0.01). However, BaSC06 does not have these functions (Figure 2B). This verifies our previous conjecture that both BaSC06 and AMP have protective effects on the host, but at present, the protective functions of the two are not synergistic and are more inclined to different aspects, since many parameters showed significant differences only after the addition of AMP. LDH is a stable cytoplasmic enzyme that possesses oxidation-reduction activities. When cells are damaged by intracellular or extracellular stress, LDH will rapidly release into the extracellular environment. ALT and AST as important hallmarks for liver damage, increased activity reflects cell damage. There were no significant differences among the three groups (*P* > 0.05), indicating that BaSC06 as well as AMP are not cytotoxic and do not damage the host liver (Figure 2C).

### Short-chain fatty acid analysis in cecum contents

**Figure 3:**
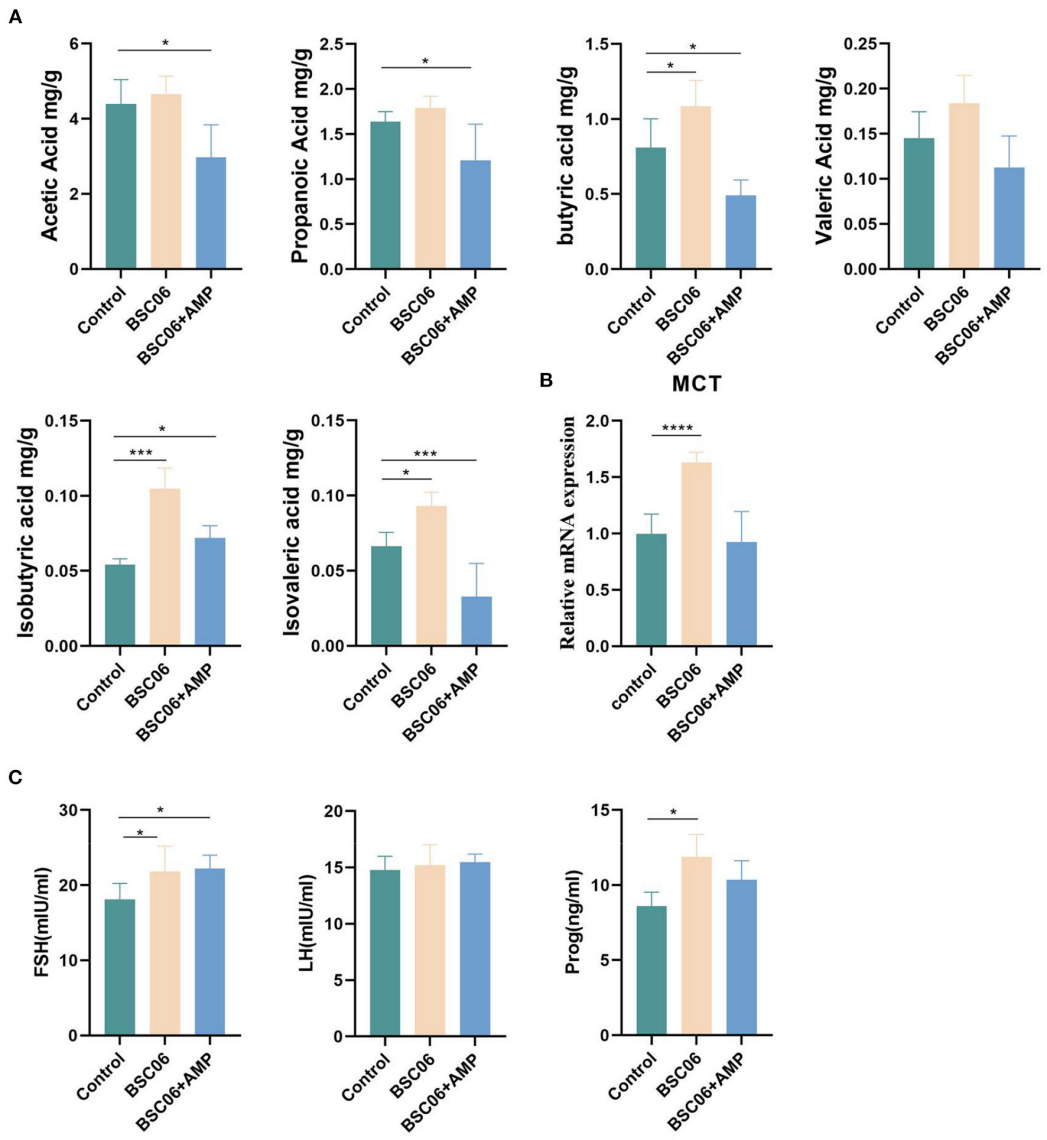
**(A)** Short-chain fatty acids in colonic contents (*n* = 5). **(B)** mRNA expression level of SCFAs recipient MCT (*n* = 4). **(C)** Sex hormone level of laying hens (*n* = 6). Data are presented as the means ± SD; *P < 0.05, ***P < 0.001, ****P < 0.0001.

SCFAs are the main players in the interplay between diet, microbiota, and health (19). BaSC06 significantly increased the content of butyric acid (*P* < 0.05), isobutyric acid (*P* < 0.001) and isovalerate acid (*P* < 0.05) in the cecum and increased the expression of MCT (*P* < 0.0001), a short chain fatty acid receptor. In contrast, the addition of AMP reduced the content of all short-chain fatty acids except isobutyric acid, compared to the control group (*P* < 0.05; Figures 3A,B). This result suggests that AMP may weaken the effect of BaSC06 by reducing the abundance of bacteria that can produce short-chain fatty acids. In order to explore how SCFA can improve egg quality, we tested the sex hormones of laying hens. Compared with the control group, the addition of BaSC06 promoted the secretion of FSH and Prog (*P* < 0.05), and the effect of BaSC06 on Prog was weakened after the addition of AMP (Figure 3C).

### Cecum microbiota analysis

#### Analysis of the number of species and quantity differences

**Figure 4:**
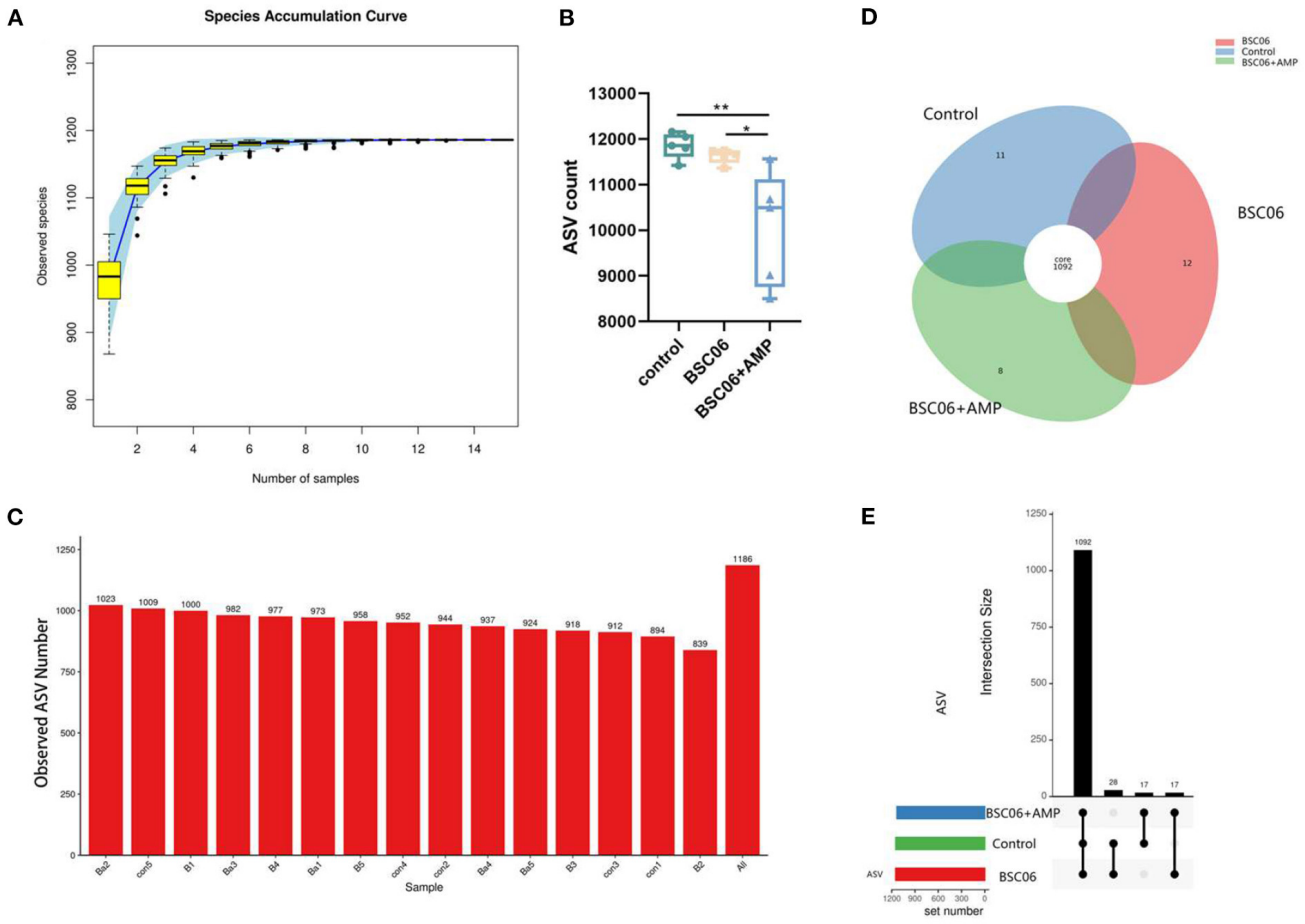
Analysis of the number of species in cecum contents. **(A)** Species accumulation curve of samples number (*n* = 5). **(B)** Number of detected ASVS counts. **(C)** Observed species number (con, control; B, *Bacillus amyloliquefaciens SC06*; Ba, *Bacillus. amyloliquefaciens SC06* combined with antimicrobial peptide). **(D,E)** Describe the number of differential ASVS between groups. *P < 0.05, **P < 0.001.

To validate our assumptions, we used sequencing of the 16S ribosomal RNA gene to investigate altered microbiome distribution in the cecal contents. As the number of samples increased, fewer species were observed and the curve flattened, indicating that we sequenced a sufficient number of samples (Figure 4A). Interestingly, we observed that the addition of AMP only significantly reduced the counts of ASVs (*P* < 0.01), but not the number of species observed. Notably, the number of ASVs reduced by AMP was much higher than the sum of any one or several species in the intestine. This suggests that the effect of AMP on the intestinal flora is widespread but not lethal, as there was no difference in the number of species observed (Figures 4B,C). In addition, 1,092 species were present in all three groups, 11 species were present only in the control group, 12 species were present only in the BaSC06 group, and 8 species were present only in the BaSC06+AMP group. Compared to the other two groups, 17 species were not observed in the control group, 17 species were not observed in the BaSC06 group, and 27 species were not observed in the BaSC06+AMP group (Figures 4D,E).

#### Analysis of bacterial taxonomic composition

**Figure 5:**
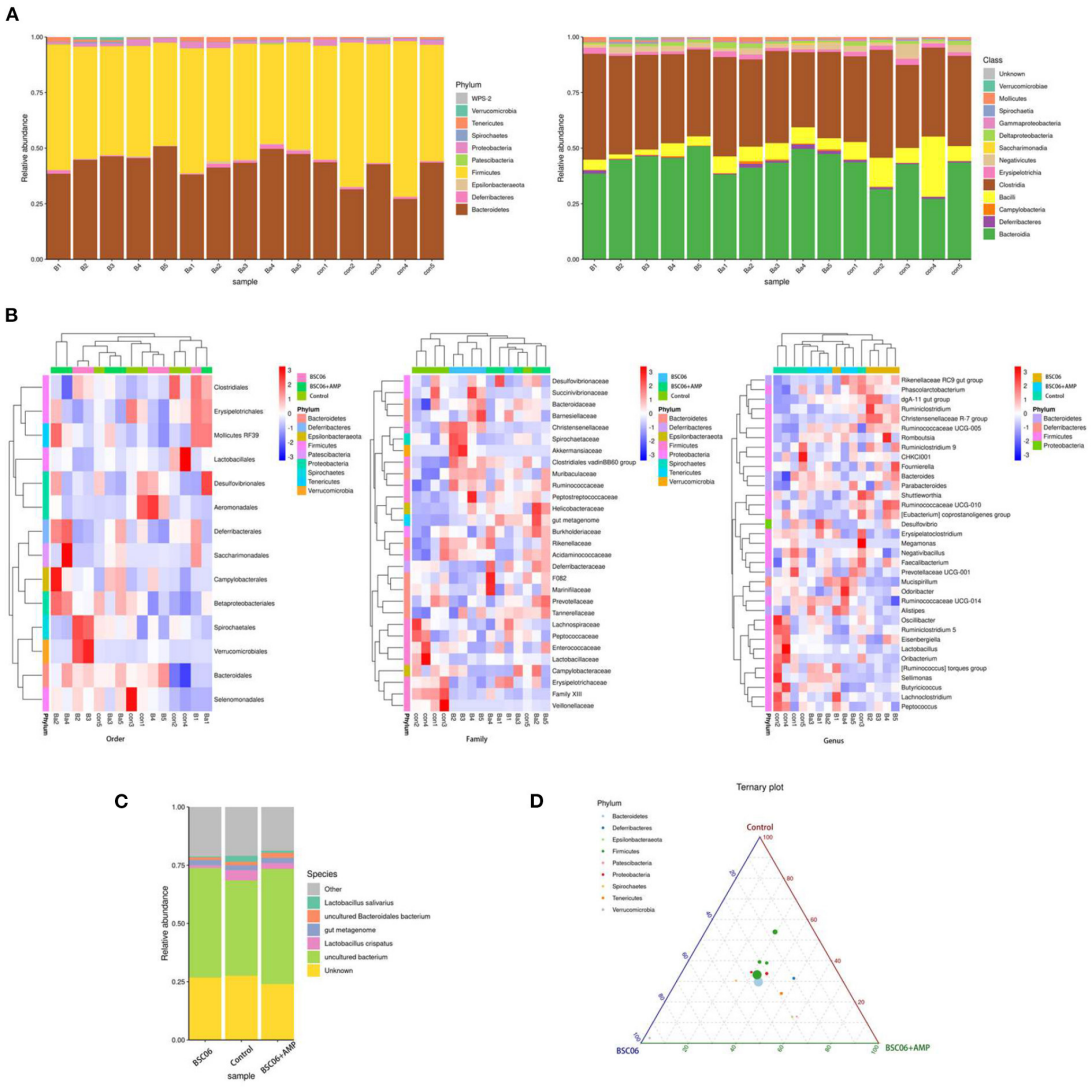
Bacterial taxonomic composition of cecum contents. **(A)** Relative abundance of phylum and class level. **(B)** Highest relative abundance in the different groups of Order, Family and Genus level. **(C)** Relative abundance of Species. **(D)** Distribution of the different class in the three groups, the size of the points represents the average abundance of the class, and the color of the points represents the phylum from which the class is derived.

As shown in Figures 5A,B, there were no meaningful differences between groups in phylum, class and order level, and the composition distribution within the Control group was not stable enough. However, at the family and genus level, intra-group differences were reduced and the same groups were clustered together. Noticeably, the compositions of the Control and BaSC06+AMP groups are more similar at the genus level compared to BaSC06. Also, we note that this similarity is mainly present in Firmicutes (Figure 5B). Distribution of the different Phylum in the three groups is shown in Figure 5D. The above results suggest that BaSC06 mainly changed the distribution of bacterial composition in some families and genera, while at the genus level, the addition of AMP moved the distribution of the flora toward the control group similarity, probably because the effect of BaSC06 on some genera with high abundances was attenuated by AMP.

#### Difference analysis of ASV at the genus level

**Figure 6:**
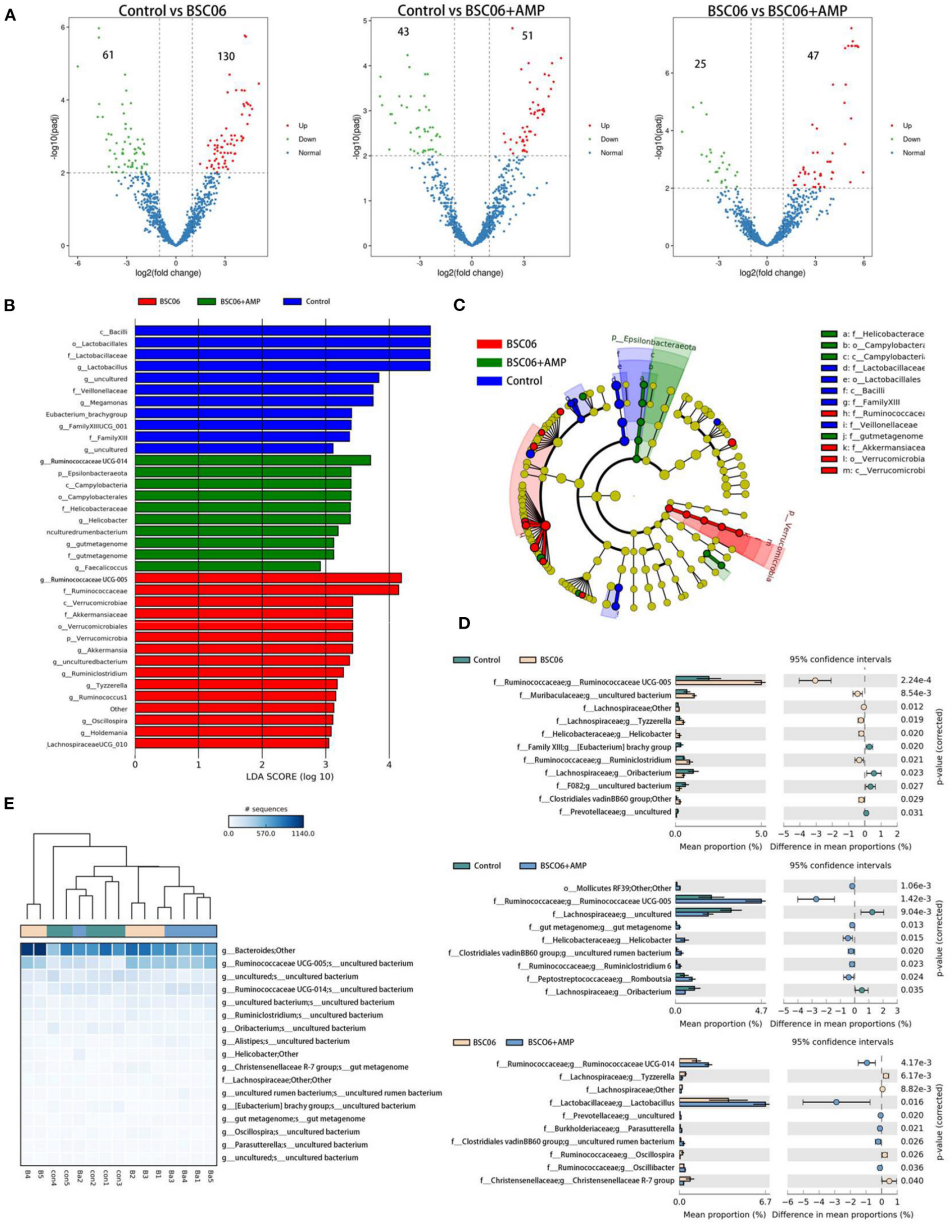
**(A)** Analysis of changes in absolute species abundance between groups using Deseq. **(B,C)** LEfSe bar and LEfSe cladogram. **(D)** Genus differences between two or three groups **(E)** were analyzed by STAMP.

Finally, analyzing the number of differential bacteria at the species level, the number of species differences between the groups is shown in Figure 6. Notably, BaSC06 significantly increased the number of species in the control group by 130, which decreased to 51 after the addition of AMP (padj < 0.001). However, most bacteria are not classified at the species level, so our subsequent analysis focused on the genus level (Figure 5C). The addition of AMP significantly reduced the count of intestinal flora in laying hens, which would affect the subsequent analysis, so we normalized the ASV counts of the three groups before analysis. Generated from the linear discriminant analysis effect size (LEfSe) analysis, showed distinct gut microbiota compositions among laying hens from all groups. The comparison of dominant bacterial taxa at the genus level suggested that BaSC06 increased the relative abundance of *Ruminococaaoeae UCG*-005 compared with the other group (*P* < 0.0001). *Ruminococcaceae_UCG-005* is a well-recognized butyrate-producing gut bacteria (20). The results of STAMP analysis showed similar results, and the abundance of *Ruminococcaceae_UCG-005* was still higher than the control after the addition of AMP (*P* < 0.0001; Figure 6D). Furthermore, the addition of BaSC06 reduced the relative abundance of lactobacillus compared to the control, but STAMP analysis showed that AMP addition reversed this trend, again demonstrating that AMP attenuates the effect of BaSC06 (Figures 6B,D). Figure 6E shows the species that are different between the three groups according to *p*-value, in contrast to Figures 5B, 6E shows a smaller abundance of species and cluster analysis shows a higher similarity between the control and BaSC06 groups, this may be due to the smaller relative abundance of species that are more sensitive to AMP. In conclusion, BaSC06 was able to increase the amount of butyric acid in the gut through an increase in the relative abundance of *Ruminococcaceae_UCG-005*, while AMP was unable to reverse this effect, suggesting that the effect of AMP may be based on a broad-spectrum reduction of gut microbial populations, consistent with previous speculation.

#### Microbiota diversity in cecum contents

**Figure 7:**
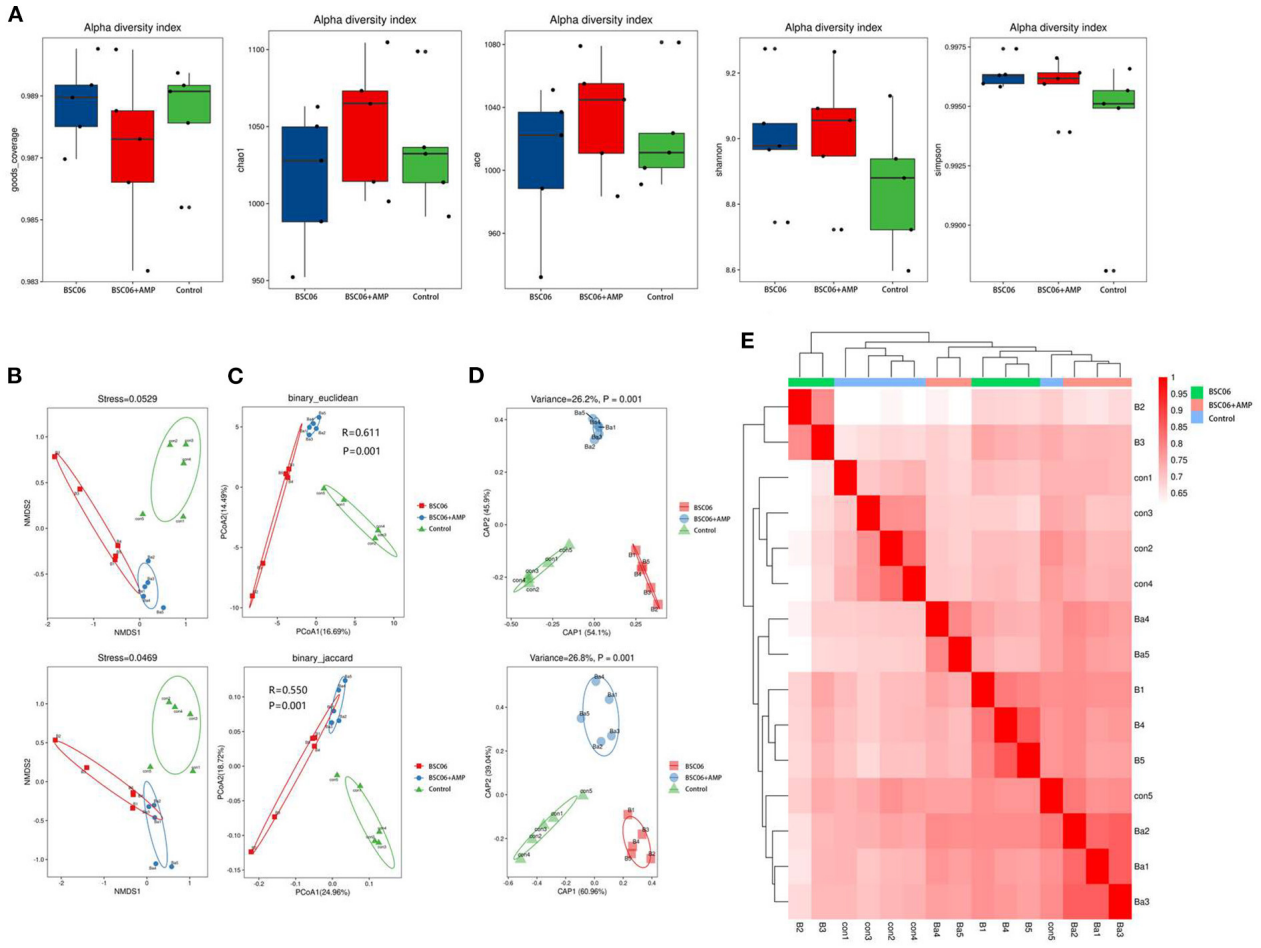
Addition of BaSC06 and AMP alters microbial diversity in the cecum contents of laying hens. **(A)** Alpha diversity. Analysis of beta diversity using. **(B)** Non-metric multidimensional scaling (NMDS). **(C)** Principal co-ordinates analysis (PCoA). **(D)** Constrained PCoA (CPCoA). **(E)** Heatmap based on binary_jaccard analysis results.

There were no significant differences among all treatments for the indices of α diversity, including the observed Goods, Chao1, ACE, Shannon, and Simpson indices of Cecum (Figure 7A). In addition, the weighted principal coordinate analysis (PCOA), non-metric multidimensional scaling (NMDS) and Constrained PCoA (CPCoA) analysis plots of cecal microbiota (Figures 7B–D) verified that there were significant (*P* < 0.05) differences in microbial communities among all treatments, indicating that the addition of BaSC06 changed the bacterial community structure in the cecum. In addition, the microbial communities of BaSC06+AMP were more similar to that of BaSC06 compared to the control. This indicates that BaSC06 in the BaSC06+AMP group still has a role in optimizing the microbiota.

#### Predicted functions in the gut microbiota

**Figure 8:**
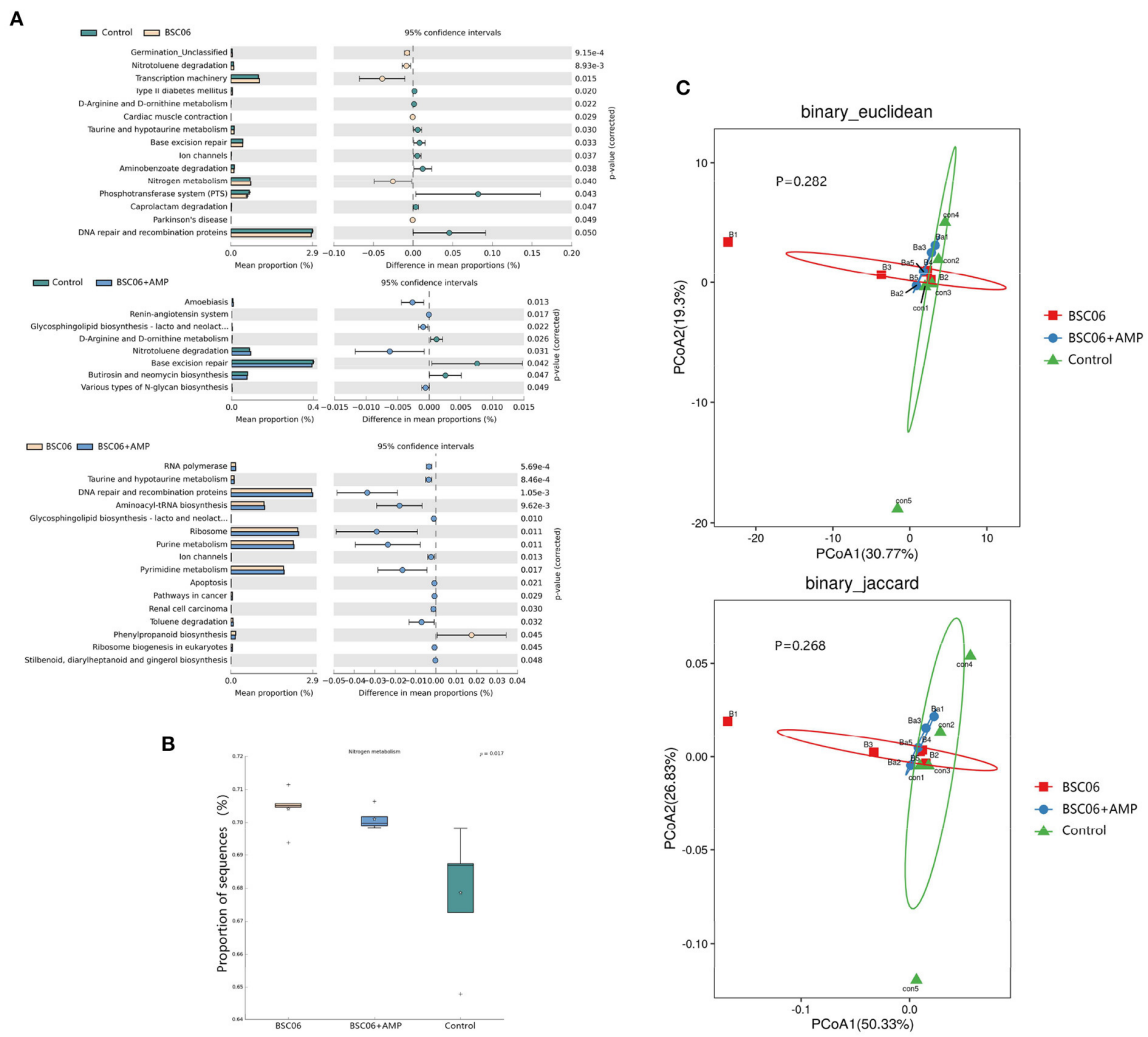
Predicted functions and diversity in the gut microbiota. **(A)** Extended error bar plot for two-group analysis module comparison of PICRUSt predicted KEGG function data using Welch's *t*-test. **(B)** Abundance of microbiota related to nitrogen metabolism. **(C)** PCoA analysis of KEGG function.

The PICRUSt approach was used to evaluate the functional potential of microbial communities. The results showed that BaSC06 and BaSC06+AMP treatment increased the abundance of microbiota related to the metabolism of D-arginine and D-ornithine metabolism and nitrotoluene degradation compared to the control treatment (Figure 8A). Birds have a unique nitrogen metabolism and excrete nitrogen as water-insoluble uric acid-therefore (21). Figure 8B shows that the addition of BaSC06 significantly increased the abundance of microorganisms associated with nitrogen metabolism, whereas only BaSC06+AMP significantly increased UA levels in the serum of laying hens. It is possible that AMP has reduced the number of bacteria which can degrade uric acid. To investigate the differences in overall gut microbial function, we performed PCoA analysis of KEGG function data from the three groups (Figure 8C). The results showed no significant differences in microbial function between the three groups and the microbial function BaSC06+AMP group had extremely similar to the control group.

#### Correlation analysis

**Figure 9:**
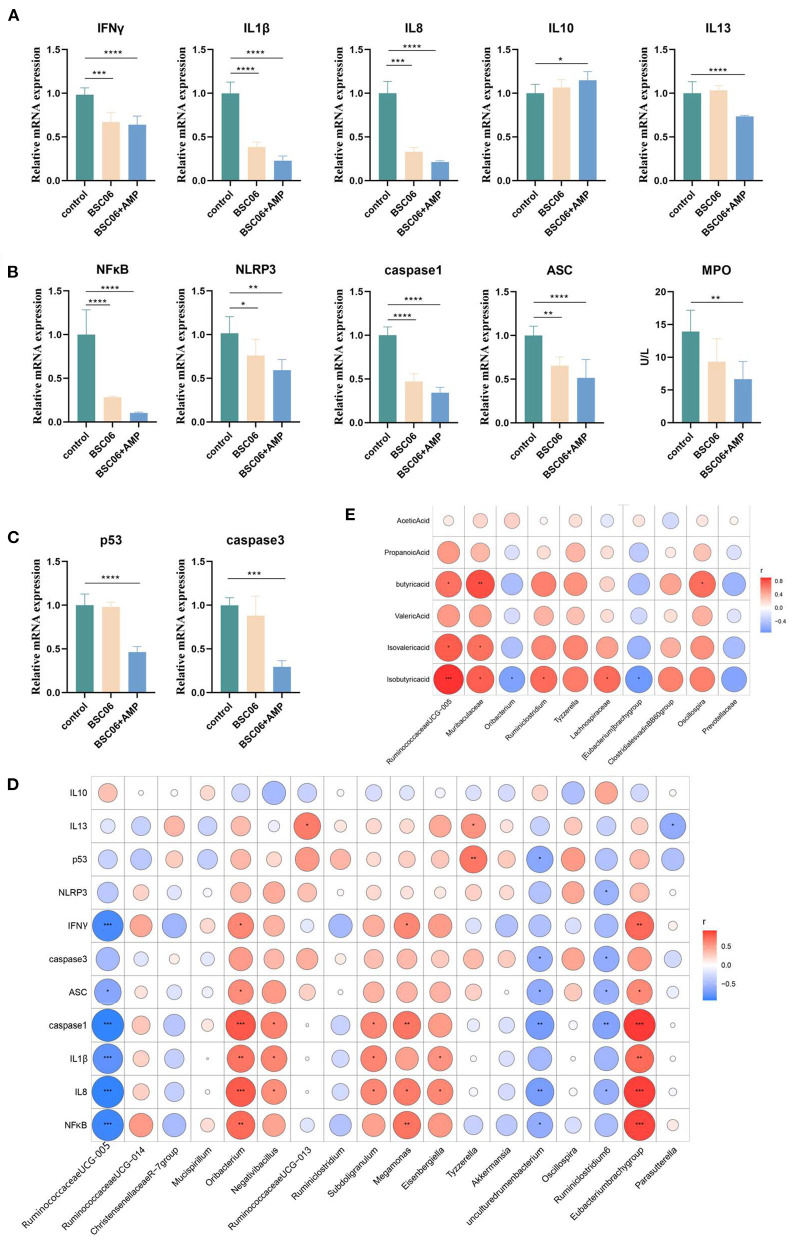
Correlation between expression of inflammation and apoptosis related genes and differential bacteria. **(A)** Relative mRNA expression of cytokines. **(B)** Relative levels of canonical activation of the inflammasome related mRNA. **(C)** Relative mRNA expression of critical proteins for apoptosis. **(D)** Pearson's correlation analysis of differential genus and gene expression. **(E)** Pearson's correlation analysis of differential genus and short-chain fatty acid. *P < 0.05, **P < 0.01, ***P < 0.001, ****P < 0.0001.

Short-chain fatty acids are closely related to intestinal immunity of host (22). To assess the effect of BaSC06 or BaSC06+AMP on host immune levels, we measured the expression levels of cytokine mRNA in the ileum. The results showed that BaSC06 significantly inhibited the expression of pro-inflammatory factors (*P* < 0.0001), meanwhile, AMP enhanced this effect and significantly increased the mRNA expression level of IL10 (*P* < 0.05; Figure 9A). It is possible that after optimizing the microbial community with BaSC06, AMP reduces the number of gut microbes, leading to a reduction bacteria-derived antigens thus reducing the level of inflammatory factors (9). Figure 9B shows the gene expression level of canonical NLRP3 inflammasome activation pathway protein, the results of which are consistent with the trend in Figure 9A, and the addition of AMP significantly reduced the level of MPO in serum (*P* < 0.01). Intestinal epithelial cell (IEC) apoptosis has increasingly been recognized to have a prominent role in ulcerative colitis (UC). Inflammatory bowel disease (IBD) has a principal role in host health and mediates the intestinal mucosal injury and epithelial apoptosis (23). Figure 9C shows the gene expression levels of critical apoptosis-related proteins, and the results show that only the addition of AMP significantly decreases the gene expression of related proteins. It indicates that the intestine of hens in the BaSC06+AMP group had the lowest inflammatory response (*P* < 0.001). Finally, we further investigated the specific relationship between inflammation- and apoptosis-related gene expression levels and differential bacteria using Spearman correlation analysis. The abundance of *Ruminococaaoeae UCG-005* and *Ruminiclostridium6* was negatively correlated with the expression of the above genes. It suggests that these bacteria may have potential anti-inflammatory effects (Figure 9D). The estimates of Mendelian randomization Egger suggested that genetically predicted *Ruminiclostridium6* was negatively associated with systemic lupus erythematosus (SLE), indicating a potential immunomodulatory role of *Ruminiclostridium6* (24). Finally, we screened for species with significantly altered absolute abundance in the control and BaSC06 groups, analyzed their correlation with short-chain fatty acids (Figure 9E). Besides *Ruminococaaoeae UCG-005*, we also found that *Muribaculaceae* and *Oscillospira* were positively correlated with butyrate. These two species are the reported probiotics, *Muribaculaceae*, which are thought to extend host lifespan, and *Oscillospira*, whose abundance was negatively associated with multiple diseases (25, 26).

## Discussion

Probiotics are live microorganisms that provide health benefits to the host when ingested in sufficient amounts (27). Our team has previously demonstrated that *B. amyloliquefaciens* SC06 is beneficial to the intestine of piglets which also has an antioxidant effect on IPEC-1 cells (8, 28). In addition, supplementing the diet with a combination of glucose oxidase and *B. amyloliquefaciens* SC06 can enhance immune function and improve the composition of the broiler's intestinal microbiota. The combined treatment of glucose oxidase with *B. amyloliquefaciens* SC06 has a stronger effect than the single component treatment (9, 29). Whereas, in our results, the hens treated with BaSC06 alone had better egg quality, the BaSC06+AMP group showed a decrease in egg quality compared to the BaSC06 group, but there was a trend toward better egg production and feed conversion ratio (Tables 1, 2). The reason for this difference may be due to the difference in short-chain fatty acid content.

Short chain fatty acids (SCFA) are the primary end products of fermentation of non-digestible carbohydrates (NDC) that become available to the intestinal microbiota (30). SCFA, especially butyrate, is an important substrate for maintenance the colonic epithelium. Butyrate is the preferred fuel utilized by colonocytes and the primary site of butyrate sequestration is the gut epithelium (31). Butyrate is an important regulator of tight junction proteins and has been shown to enhance intestinal barrier function through increased expression of claudin-1, Zonula Occludens-1 (ZO-1) and occludin redistribution, proteins which are components of the tight junction assembly (32). The highest levels of butyrate were detected in the BaSC06 group, suggesting that influencing host intestinal health through butyrate is one of the pathways through which BaSC06 exerts its effects (Figure 3A). Evidence exists for butyric acid exerting beneficial effects on the laying and hatching performances of hens (33, 34). The improvement in laying performance and egg quality in the BaSC06 group may be attributed to the fact that *B. amyloliquefaciens* SC06 increased the amount of butyrate in the cecum. The addition of AMP decreased the content of butyrate in the cecum, even lower than in the control group, which may be the reason why the egg quality of the BaSC06+AMP group was not as good as that of the BaSC06 group.

Cecropins reduced the number of aerobic bacteria in the broiler jejunum and feces in a dose-dependent manner, while increasing the height of the intestinal villi (35). Similar to our results, the BaSC06+AMP group had the longest jejunal microvilli and the lowest number of detected bacteria (Figures 1B,C, 4B). *Bacillus amyloliquefaciens* SC06 significantly increased the number of *Ruminococcaceae_UCG-005*, a well-recognized butyrate-producing gut bacterium in the cecum. Different from *B. amyloliquefaciens* SC06, we believe that the main improvement of AMP on production performance is in the maintenance of intestinal health, because AMP reduces the content of butyrate in the cecum by reducing the number of intestinal microorganisms (Figures 3A, 4B). Moreover, the addition of AMP significantly increased the antioxidant enzyme activity in the serum of laying hens (Figure 2B). All these results indicate that the addition of AMP is beneficial to the health of laying hens. However, this does not indicate that only AMP was functioning in the BaSC06+AMP group. We observed an elongation of jejunal microvilli in the BaSC06 group as well, and the GSH-Px activity was not significantly different from that of the BaSC06+AMP group (Figures 1B, 2B), and most importantly, the microbiota of the BaSC06 and BaSC06+AMP groups had a high similarity compared to the control group (Figure 7). Therefore, we suggest that *B. amyloliquefaciens* SC06 first altered the gut microbiota of laying hens and subsequently AMP reduced the absolute abundance of several species on this basis. As previously mentioned, the number of ASVs reduced by AMP was much higher than the sum of any one or several species in the intestine, this indicates a wide range of AMP action, which is consistent with the effects of cecropins (14). The effect of AMP on SCFA is closely related to its species. Supplementation of Melittin and Cecropin A in the diet of rats has been reported to significantly reduce the concentration of SCFA in the feces (36). Microcin C7 significantly increased the levels of lactic acid and acetic acid in the ileum and cecum of broiler chickens, but Microcin C7 significantly decreased the concentration of butyric acid at 6 mg/kg (37). Especially in pathological conditions, Cathelicidin-WA can increase the SCFA concentration in the feces of weaned piglets by increasing the number of Lactobacillus (38).

Inflammation is a host response defined by the infiltration of immune cells into affected tissues. It has a central role in mediating host defense against pathogens, tissue repair, and restoration of homeostasis (39). Pro-inflammatory cytokines IL-1β have critical roles in tissue homeostasis in the intestinal epithelium, their expression is controlled by the inflammasome (40). There are important evidences regarding the involvement of NLRP3 inflammasome in different inflammatory diseases such as cerebral ischemia, Parkinson's and Alzheimer's diseases, inflammatory bowel disease and atherosclerosis (41). NLRP3 is a group of high molecular weight cell membrane protein complexes formed to mediate the host immune response to several damage-associated molecular patterns (DAMPs) and pathogen-associated molecular patterns (PAMPs). This complex consists of three major parts: (1) a sensor/receptor protein that serves as a platform for complex formation at cell membrane sites, such as NLRP3; (2) an ASC (apoptosis-associated speck-like protein containing CARD) adapter protein; (3) an effector protein, pro-caspase-1 (42). After conformational of inflammasome activation, NLRP3 interacts with the ASC adapter protein to induce ASC aggregation into a large cytosolic protein speck. Then, ASC specks generate a platform for recruitment of pro-caspase-1 monomers, which promotes its self-cleavage and activation. Consequently, active caspase-1 stimulates the cleavage of pro-IL-1β into mature IL-1β, thus promoting inflammatory responses (43). *Bacillus amyloliquefaciens* SC06 reduces inflammation by improving the intestinal microbiota composition of broilers (29). In our results, *B. amyloliquefaciens* SC06 reduced gene expression of critical genes on the NLRP3/caspase-1/IL-1 axis, leading to reduced gene expression of IL1β, ultimately acting as an anti-inflammatory agent. This effect should be attributed to the increased content of short-chain fatty acids and the improvement of the intestinal microbiota composition. The addition of AMP increased the anti-inflammatory effect, because the microbiota of the BaSC06+AMP group was similar to that of BaSC06 and reduced the number of intestinal microorganisms in the presence of AMP. The combined effect of the two active components greatly alleviated the host immune response (Figures 9A,B).

In conclusion, our results demonstrate that BaSC06 promotes the health of laying hens by increasing the content of short-chain fatty acids and improving the intestinal microbiota composition, ultimately improving production performance and egg quality. However, the addition of AMP reduced the number of gut microorganisms leading to a decrease in the content of short-chain fatty acids, while attenuating immune response simultaneously, this could be the reason why the quality of eggs in the BaSC06+AMP group was not as good as in the BaSC06 group.

## Conclusion

In general, BaSC06 can increase the content of butyrate in the cecum by changing the gut microbiota and improving the abundance of butyrate-producing bacteria, which finally improves the lying performance and egg quality of laying hens. When BaSC06 was used together with AMP, AMP reduced the abundance of bacteria based on the alteration of the gut microbiota by BaSC06, thus reducing the content of short-chain fatty acids but alleviating the immune response.

## Data availability statement

The datasets presented in this study can be found in online repositories. The names of the repository/repositories and accession number(s) can be found below: https://www.cncb.ac.cn/, GSA: CRA007386.

## Ethics statement

The animal study was reviewed and approved by Institutional Animal Care and Use Committee of Zhejiang University.

## Author contributions

SX: conceptualized the experiments, performed the experiments, analyzed the data, and wrote the original draft. FW and PZ: performed part of the experiments. XL, QJ, QW, BW, YZ, LT, and DY: revised the manuscript. WL: conceptualized the experiments and revised the manuscript. All authors contributed to the article and approved the submitted version.

## Funding

This study was supported by the National Natural Science Foundation of China (No. 32072766).

## Conflict of interest

The authors declare that the research was conducted in the absence of any commercial or financial relationships that could be construed as a potential conflict of interest.

## Publisher's note

All claims expressed in this article are solely those of the authors and do not necessarily represent those of their affiliated organizations, or those of the publisher, the editors and the reviewers. Any product that may be evaluated in this article, or claim that may be made by its manufacturer, is not guaranteed or endorsed by the publisher.
